# Interpretable Machine Learning for Prediction of Post-Fire Self-Healing of Concrete

**DOI:** 10.3390/ma16031273

**Published:** 2023-02-02

**Authors:** Magdalena Rajczakowska, Maciej Szeląg, Karin Habermehl-Cwirzen, Hans Hedlund, Andrzej Cwirzen

**Affiliations:** 1 Department of Civil, Environmental and Natural Resources Engineering, Luleå University of Technology, 971 87 Luleå, Sweden; 2 Faculty of Civil Engineering and Architecture, Lublin University of Technology, 40 Nadbystrzycka Str., 20-618 Lublin, Poland; 3 Skanska Sverige AB, 405 18 Göteborg, Sweden

**Keywords:** autogenous self-healing, cementitious materials, high temperature, artificial neural network, ensemble methods, mechanical properties, artificial intelligence

## Abstract

Developing accurate and interpretable models to forecast concrete’s self-healing behavior is of interest to material engineers, scientists, and civil engineering contractors. Machine learning (ML) and artificial intelligence are powerful tools that allow constructing high-precision predictions, yet often considered “black box” methods due to their complexity. Those approaches are commonly used for the modeling of mechanical properties of concrete with exceptional accuracy; however, there are few studies dealing with the application of ML for the self-healing of cementitious materials. This paper proposes a pioneering study on the utilization of ML for predicting post-fire self-healing of concrete. A large database is constructed based on the literature studies. Twelve input variables are analyzed: w/c, age of concrete, amount of cement, fine aggregate, coarse aggregate, peak loading temperature, duration of peak loading temperature, cooling regime, duration of cooling, curing regime, duration of curing, and specimen volume. The output of the model is the compressive strength recovery, being one of the self-healing efficiency indicators. Four ML methods are optimized and compared based on their performance error: Support Vector Machines (SVM), Regression Trees (RT), Artificial Neural Networks (ANN), and Ensemble of Regression Trees (ET). Monte Carlo analysis is conducted to verify the stability of the selected model. All ML approaches demonstrate satisfying precision, twice as good as linear regression. The ET model is found to be the most optimal with the highest prediction accuracy and sufficient robustness. Model interpretation is performed using Partial Dependence Plots and Individual Conditional Expectation Plots. Temperature, curing regime, and amounts of aggregates are identified as the most significant predictors.

## 1. Introduction

The popularity of concrete as a building material stems from its high compressive strength and versatility for structural applications [1,2]. Unfortunately, it is also a brittle material that loses durability due to cracking caused by harsh environmental conditions [3], e.g., high-temperature exposure [4]. On the other hand, cementitious materials have an intrinsic auto-repair capacity, called autogenous self-healing, which enables them to seal cracks and leads to their improved durability and recovered mechanical properties [5]. Concrete can also auto-repair damage after exposure to high temperatures, e.g., fire [6], the efficiency of which depends on multiple factors: maximum temperature loading [7], mix composition [8,9], and the application of post-fire cooling and curing [10,11,12]. During a fire, concrete undergoes physical and chemical transformations, e.g., induced thermal stresses and the evaporation of free water cause severe cracking [10]. Therefore, effective post-fire self-healing properties are a benefit that would increase the likelihood of concrete reuse in the event of a fire. Regarding sustainability, concrete structures with self-healing properties are an environmentally friendly option with less material needed for repair, resulting in decreased carbon emissions and lower costs [5,13].

Accurate prediction of concrete’s self-healing, including its post-fire recovery behavior, is of paramount importance as it would reduce costs of expensive destructive testing and give an indication of the performance of “intelligent” cementitious materials. Unfortunately, there were few attempts to model concrete’s post-fire auto-repair [14]. An analytical model of the post-fire recovery of mechanical properties was proposed based on stress–strain curve fitting [14]. Nevertheless, the model was applicable only to cement-lime mortars. A multi-scale model was developed based on the physicochemical principles, considering carbonation, decarbonation, and recarbonation processes [15]. A successful prediction was obtained for the compressive and tensile strength recovery after high-temperature loading; however, the complexity of the model was high. Concrete’s post-fire behavior was also included in the current American Concrete Institute (ACI) and Eurocode standards [16,17], yet, cooling or curing regimes were not considered [18]. Hence, it is necessary to develop new models to facilitate designing post-fire self-healing materials, analyzing their performance, and predicting the probability of recovery.

In the past decades, machine learning (ML) methods have been successfully applied in the modeling of cementitious materials. ML algorithms enable the analysis of multiple variables and the processing of large amounts of data [19]. ML techniques have proved to be successful in predicting the mechanical properties of concrete, e.g., compressive strength [20,21], tensile strength [22,23], and elastic modulus [24] Furthermore, ML is efficiently applied in analyzing durability and deterioration processes, e.g., sulfate attack [25], chloride diffusion [26], and alkali–silica reaction [27]. Recently, applications in more sophisticated areas were proposed, e.g., predicting hydration kinetics [28]. 

One of the significant drawbacks of ML modeling is a lack of interpretability [29] leading to their limited applicability. Many ML models follow the so-called “black box” approach, i.e., they exhibit high complexity and excellent prediction capacity, but they are difficult to explain. Interpretability is essential because it facilitates finding patterns in trained models and errors in less accurate predictions [30]. Several interpretation methods have been proposed, including Partial Dependence Plots [31] and Individual Conditional Expectation Plots [32]. These methods make it possible to study the response’s dependency on the predictor variables and formulate a causal explanation of the ML “black box”.

Despite the popularity in other areas of concrete science, ML techniques are rarely used to model the autogenous self-healing properties of concrete (Table 1). A recent study proposed a mixed approach, utilizing meta-analysis and Artificial Neural Networks (ANN) to evaluate the relationship between, e.g., mix composition and the self-healing index [33]. In contrast to most studies, the self-healing output was a combination of durability and mechanical performance recovery. Nevertheless, limited accuracy was obtained, with a coefficient of determination (R^2^) of approximately 0.77 for the validation and testing set [33]. The auto-repair capability of engineered cementitious materials was successfully modeled with ensemble methods, i.e., AdaBoost, bagging, and stacking, to increase prediction accuracy. Good precision was achieved, with R^2^ greater than 0.85; however, only four input variables were employed: initial crack width, fly ash, silica fume, and hydrated lime powder [34]. Six different ML algorithms, i.e., ANN, k-nearest neighbors, decision tree regression, Support Vector Regression, and two ensemble models (gradient boosting regression and Random Forest) were trained on an extensive database of more than 1400 records to predict autogenous self-healing of concrete with very high accuracy [35]. Sixteen predictors were included: type and dosage of healing material; fiber diameter, length, and tensile strength; the initial cracking data and initial cracking width; the time for healing; the healing condition (environmental exposure); the amount and type of cement; the amount of superplasticizer; fine aggregates; fly ash; slag; and the water–binder ratio [35]. The ensemble model achieved the highest coefficient of determination (R^2^) equal to 0.958. To the authors’ knowledge, there is no ML-based model of post-fire self-healing. In addition, the autogenous self-healing ML models focus primarily on prediction accuracy, paying less attention to sensitivity analysis and model interpretation, which indicates a significant research gap in this area.

The objective of this paper was to develop an interpretable ML model for predicting the post-fire recovery of the compressive strength of concrete. A detailed database with 197 records was prepared based on the available literature. Twelve input variables were determined: w/c, age of concrete, amount of cement, fine aggregate, coarse aggregate, peak loading temperature, duration of peak loading temperature, cooling regime, duration of cooling, curing regime, duration of curing, and specimen volume. Four ML approaches with basic architecture were evaluated, i.e., Artificial Neural Networks (ANN), Support Vector Machines (SVM), Regression Trees (RT), and an Ensemble of Regression Trees (ET). The final model was chosen based on four statistical measures, i.e., mean squared error (MSE), root mean squared error (RMSE), mean absolute error (MAE), and the coefficient of determination (R^2^). In addition, Partial Dependence Plots (PDP) and Individual Conditional Expectation (ICE) Plots were used to study the effect of different predictors on the response.

## 2. Research Significance

The design of materials adheres to the path paved by four principal elements: processing, structure, properties, and performance (PSPP) [38]. Science aims to understand how the materials’ performance depends on their processing and structure; it is the so-called “forward” approach. Based on the results of scientific experiments, the forward models, which facilitate the prediction of the material’s properties and performance, can be formulated [39]. Furthermore, these models can be used to construct “inverse” problems, enabling, e.g., optimization of the material structure to achieve the desired performance, which is crucial from the engineering point of view. Data-driven modeling, such as machine learning, facilitates the creation of both forward (e.g., [21]) and inverse models (e.g., [40]). In concrete science, developing accurate models is essential since concrete is a complex heterogenous material with infinite composition combinations, making the experimental testing cumbersome and costly. Application of ML for the prediction of concrete properties, similar to that attempted in this study, could facilitate optimization of the material properties, as well as the design of potential future experimental campaigns. Considering that concrete, after water, is the second-most-used substance in the world [41] and the most popular building material, the practical implication of this research is indubitable.

The novelty of this study involves a pioneering application of ML for the prediction of self-healing of thermally cracked concrete. The literature review indicates that there were no attempts to model the post-fire self-healing of concrete using artificial intelligence. Therefore, the current study fills the research gaps through the following points: (i) the paper compares several optimized ML methods, i.e., ANN, SVM, RT, and ET, with linear regression, with the selected trained model with the best accuracy (ET) providing a very good correlation (exceeding 90%) with the experimental data; (ii) a robustness analysis is conducted with the use of Monte Carlo simulations to compare the stability of the selected models; (iii) sensitivity analysis is performed by calculating the PDP and ICE plots, showing the importance of the input variables with respect to the self-healing performance.

## 3. Database Description

In ML modeling, database transparency and data quality are critical [42]. At the same time, the number of observations analyzed should be at least one order higher than the number of variables [19]. Therefore, in this study, an extensive database was established based on the current state-of-the-art on post-fire self-healing of concrete to maximize the number and representativeness of the data. 

The database was constructed by combining several smaller experimental datasets available in the literature (Table 2).

In total, the database consisted of 197 records obtained from 12 experimental studies. The maximum number of samples was equal to 56 from [43] and the minimum number of samples was equal to 2 from [10]. The inclusion of several datasets has its limitations. For example, raw materials used for concrete preparation, e.g., grading of aggregate, the process and technology of specimen preparation and handling, or curing conditions, may differ from study to study. Nevertheless, ML modeling requires big data and since testing of concrete is expensive and time-consuming, using results from existing research is a common practice for ML predictions of concrete properties [51,52].

In this study, the output of the model is the compressive strength recovery (*CSR*) due to self-healing, which can be defined as follows:

(1) $$CSR=\frac{\sigma_{h}}{\sigma_{0}}\;\;\;\;\left[-\right]$$

where $\sigma_{h}$ is the compressive strength after the healing process and $\sigma_{0}$ denotes the compressive strength of the intact specimens before the temperature loading.

**Figure 1:**
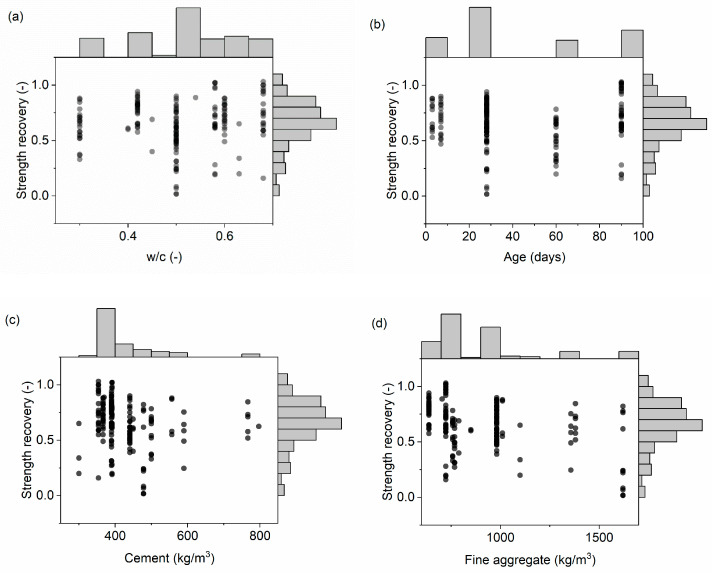

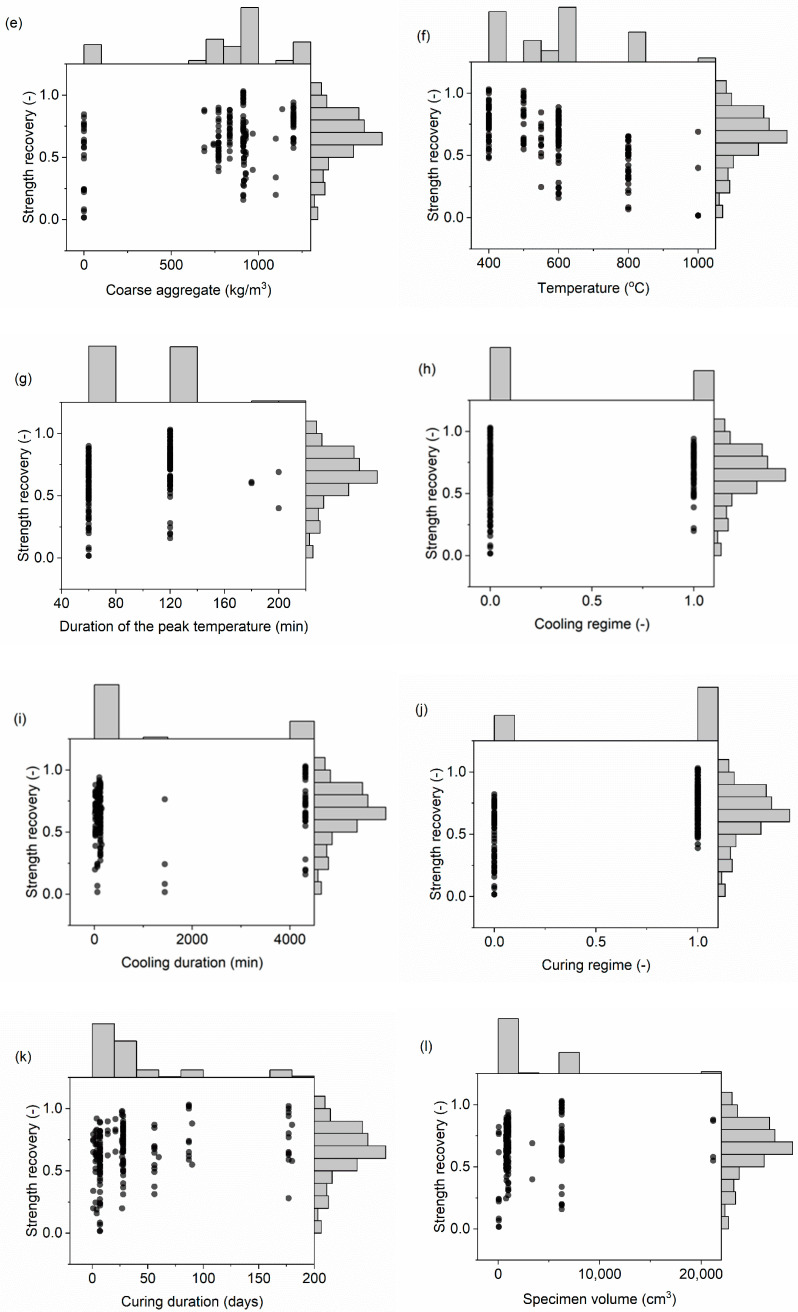
Relationship between the input and output variables, including histograms: (**a**) water-to-cement ratio (I1), (**b**) age (I2), (**c**) cement amount (I3), (**d**) fine aggregate (I4), (**e**) coarse aggregate (I5), (**f**) temperature (I6), (**g**) duration of peak temperature (I7), (**h**) cooling regime (I8), (**i**) cooling duration (I9), (**j**) curing regime (I10), (**k**) curing duration (I11), (**l**) specimen volume (I12).

Based on the analyzed literature, 12 input variables were selected as factors potentially affecting the strength recovery due to the self-healing process: w/c, age of concrete, amount of cement, fine aggregate, coarse aggregate, peak loading temperature, duration of peak loading temperature, cooling regime, duration of cooling, curing regime, duration of curing, and specimen volume. Specimen volume was calculated based on the specimen type used for compressive strength testing. When not specified, the cooling time was assumed to be 120 min based on the data reported in several studies. Several datasets were excluded from the database due to missing components, i.e., when no compressive strength was reported, and when the curing process was absent (only cooling was applied). In addition, samples with fibers and supplementary cementitious materials were omitted due to the small amount of data. Statistical descriptors of each variable, including minimum (Min) and maximum (Max) values, median, mean, standard deviation (Std), and skewness (Sk) are listed in Table 3. The histograms and relationship of each input to the output are presented in Figure 1.

Two categorical variables were assumed: cooling (I8) and curing regime (I10). Since most of the literature does not detail curing and cooling conditions, e.g., the air’s relative humidity (RH), a simplification was applied to construct the current database. In both cases, two environmental conditions were assumed, air or water, encoded by values 0 and 1 in the database, respectively.

**Figure 2:**
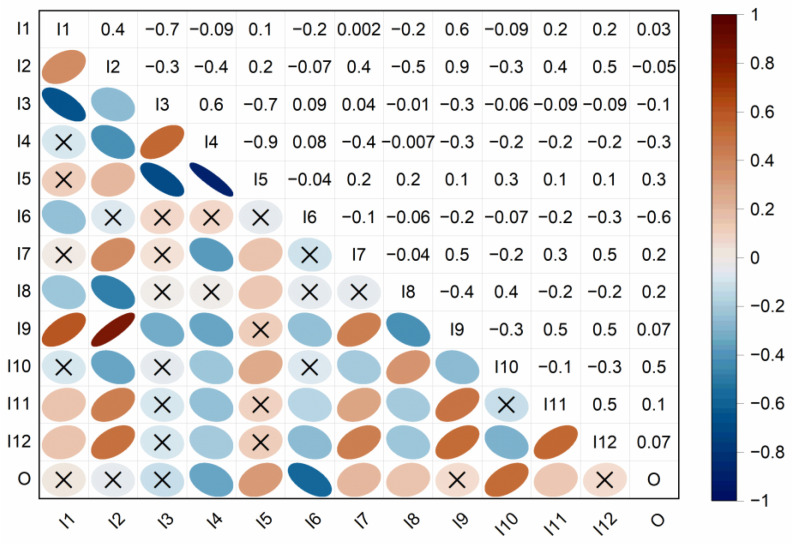
Correlation matrix of the input and output variables (x—statistically insignificant correlation).

Correlation analysis of the variables indicated strong correlations (R ≥ 0.7) between several input variables (Figure 2). 

Concrete mix composition parameters were found to be correlated, i.e., w/c (I1) and cement amount (I3) (R = −0.7), cement mount (I3) and coarse aggregate (I5) (R = −0.7), and fine aggregate (I4) and coarse aggregate (I5) (R = −0.9). In addition, a strong correlation (R = 0.9) was found between the age (I2) and cooling duration (I9) variables.

It is known that for the regression analysis, e.g., ordinary least squares regression models, the independence of the observations is assumed [53]. Nevertheless, despite the presence of correlations between several input variables, all inputs are considered for the modeling stage to perform a complete model interpretation. A similar approach was conducted by [51].

## 4. Methods

### 4.1. ML Approaches

#### 4.1.1. Support Vector Machines (SVM)

A Support Vector Machine, developed by Vapnik based on statistical learning theory [54], is a widely used supervised learning algorithm within the machine learning domain to solve classification and regression problems [54]. The intended output of SVM is the optimal n−1 subspace of the n-dimensional vector space, known as a hyperplane, that generates the largest margins between the different classes’ boundary points [55]. Kernel functions are used to evaluate the data points by calculating the higher-dimensional relationship between them as they become linearly separable [56]. Support Vector Regression (SVR) can be used to make predictions for continuous datasets, where Vapnik’s ε-insensitive loss function is used to determine the decision boundary [54,57]. SVM-based models can predict values for previously unseen data with high accuracy while being less resource intensive in terms of computation complexity.

#### 4.1.2. Regression Tree (RT), Ensemble of Trees (ET)

A Regression Tree (decision tree) is a supervised learning algorithm that can be used both for classification and regression problems. It is a popular algorithm due to its easy-to-interpret output that is presented in a tree-like, hierarchical structure. The main elements of a decision tree are the starting root point, interconnecting nodes, and single termination points, known as leaves [58].

An ensemble Regression Tree, alternatively called a Random Forest, is a compilation of multiple instances of decision trees. Consequently, it is also a supervised learning technique that can be applied to classification and regression tasks. The predictive performance increases compared to a single Regression Tree, utilizing a democratic voting process, reducing the variance [56]. However, the tradeoff is the requirement of significantly higher computational resources and harder interpretability.

Ensemble methods combine several independent learners to make an overall more robust prediction compared to an individual model. The two general types of ensemble learning techniques are bagging and boosting, where the former reduces variance and the latter reduces bias. Bagging, or bootstrap aggregating, provides a prediction based on the average of the combined predictions of all learners in the model [59]. On the other hand, boosting, e.g., LSBoost algorithm [60], iteratively adjusts its hyperparameters to compensate for the error in the previous learners’ prediction [56].

#### 4.1.3. Artificial Neural Networks (ANN)

An Artificial Neural Network is a machine learning algorithm based on and modeled after the human brain [61]. It is a supervised learning technique that is also extensively used for classification and regression problems. ANNs consist of multiple layers, such as input, hidden, and output layers, connected by neurons via axons. Each neuron represents an input to the next process step, while the axon represents the weight and biases of the associated input to the next layer. The more layers, the more complex the network. Neural networks with many layers are considered a separate field of machine learning called Deep Learning [56].

### 4.2. Performance Indices of Models

In this study, the mean squared error (MSE), mean absolute error (MAE), coefficient of determination (R^2^), root mean squared error (RMSE), and normalized root mean squared Error (NRMSE) are used as performance indices of the models. The coefficient of determination (R^2^) achieves values from 0 to 1, with higher values indicating better prediction accuracy. On the other hand, low values of MSE, MAE, and RMSE validate good precision of the model.

Performance indices of models were calculated according to the following equations:

(2) $$\mathrm{MSE}=\frac{\sum_{i=1}^{n}{\left(t-y\right)}^{2}}{n}\;\;\;\;\left[-\right]$$

(3) $$\mathrm{RMSE}=\sqrt{\frac{\sum_{i=1}^{n}{\left(t-y\right)}^{2}}{n}}\;\;\;\left[-\right]$$

(4) $$\mathrm{MAE}=\frac{\sum_{i=1}^{n}\left|t-y\right|}{n}\;\;\;\left[-\right]$$

(5) $$R^{2}=1-\frac{\sum_{i=1}^{n}{\left(t-y\right)}^{2}}{\sum_{i=1}^{n}{\left(t-\bar{t}\right)}^{2}}\;\;\;\left[-\right]$$

(6) $$\mathrm{NRMSE}=\frac{\mathrm{RMSE}}{\bar{t}}\cdot 100\;\;\;\;\left[\%\right]$$

where *n* is the number of data points, *t* is the measured (target) value for the *i*-th specimen, *y* is the predicted value from the model for the *i*-th specimen, and $\bar{t}$ is the mean value from the measured data.

### 4.3. Monte Carlo Simulations

Monte Carlo simulations (MCS) are used to assess the robustness of the models. The MCS is a sampling-based methodology introduced by [62]. It involves performing many simulations of the same process to estimate the mean. It is based on the “law of large numbers”, which states that the average of the large samples converges to the expected value μ when the number of samples n→∞. 

In this study, the sampling method of the training and testing dataset is randomized for selected models. Afterward, 800 simulations are performed with a different dataset division for training and testing. Finally, t normalized statistical convergence *C*(*N*) is calculated according to the following formula [63]:

(7) $$C\left(N\right)=\frac{1}{\bar{X}}\frac{1}{N}\overset{N}{\underset{i=1}{\sum }}X_{i}$$

where $\bar{X}$ is the mean value of the considered variable *X* and *N* is the number of Monte Carlo simulations [63].

### 4.4. Model Interpretation

The purpose of regression analysis is to find the relationship between the input variables *X* and the response *Y*, which can be described with the following equation [64]:

(8) Y=f(X,∈)

where the function *f* is the “law of nature” or the so-called “black box” and ∈ is the random noise [64]. The ML algorithms produce a non-linear, high-dimensional function *g*(*X*) which approximates function *f*(*X*) very well, making predictions of *Y* with maximum accuracy [64]. Nevertheless, due to the complexity of the ML models, their interpretation is often cumbersome, making it challenging to analyze the parameters of the model and the input variables’ importance.

Calculating the importance of each predictor by evaluating their contribution to the model’s accuracy is not the only purpose of this study. The goal is to obtain a causal interpretation of the model to understand the “law of nature”, i.e., the mechanism of post-fire self-healing, by verifying how changes in each input variable affect the changes of the response when the other variables are fixed. In this paper, the causal interpretation is applied with the use of Partial Dependence Plots (PDPs) [31] and Individual Conditional Expectation (ICE) Plots [32].

PDPs describe the average partial relationship between the input variables and the response over the marginal distribution. For linear regression, the PDPs are linear functions for each predictor. In the case of regression analysis, the average partial dependence function ${\hat{f\;}}_{x_{s}}$ on the subset of input variables $x_{s}$ can be defined with Equation (9) [31,51]:

(9) $${\hat{f\;}}_{x_{s}}\left(x_{s}\right)=\int \hat{f}\left(x_{s},x_{c})dP(x_{c}\right)$$

where $x_{s}$ is the input variable under investigation and $x_{c}$ are the other predictors from the model of function $\hat{f}$, such as $x_{s}\cup x_{c}=S$ and $dP\left(x_{c}\right)$ is the marginal effect of $x_{c}$ [31,51]. When the training dataset $\left\{S_{i},\;i=1,2,\ldots ,\;n\right\}$ is considered, the ${\hat{f\;}}_{x_{s}}$ can be calculated according to Equation (10) [65]:

(10) $${\hat{f\;}}_{x_{s}}\left(x_{s}\right)=\frac{1}{n}\overset{n}{\underset{i=1}{\sum }}\hat{f}\left(x_{s},x_{i,c}\right)$$

where $x_{i,c}$ is the actual value of the *i*-th variable in the training set and *n* is the total number of samples [65].

A PDP demonstrates the relationship between the average response and particular input. Nevertheless, in the case of strong dependencies of the analyzed variable on the other predictors, PDP can give confusing results; therefore, ICE plots can be an alternative [32]. ICE shows the functional relationship for a single observation [66]. The ICE plots display heterogeneity of the $\hat{f}$. When there is no influence between the $x_{s}$ and $x_{c}$, the curves on the ICE plot are on top of each other. However, when the relationship between $\hat{f}$ is affected by $x_{c}$, the curves will differ [32].

## 5. Modeling Sequence

All ML modeling, Monte Carlo analysis, PDP, and ICE plot calculations were performed in MATLAB software, version R2022b (Mathworks, Natick, MA, USA). Parts of the statistical analysis were performed using OriginPro, version 2021 (OriginLab Corporation, Northampton, MA, USA). Scientific color maps [67,68] were used for data visualization. 

The modeling sequence comprised the following stages.

Stage 1. Data preparation

The database containing 197 data points was split into two sets: for training and validation, 85% (167 records), and for testing, 15% (30 records). The testing data points were randomly chosen from the dataset in the beginning and fed separately to the trained and validated model. The 12 input variables were: w/c, age of concrete, amount of cement, fine aggregate, coarse aggregate, peak loading temperature, duration of peak loading temperature, cooling regime, duration of cooling, curing regime, duration of curing, and specimen volume. The output variable was compressive strength recovery.

Stage 2. Model optimization and performance assessment

Four ML approaches were analyzed, i.e., SVM, RT, ET, and ANN. Each algorithm was optimized with the hyperparameters listed in Table 4 to obtain a minimum MSE. The k-fold cross-validation algorithm was used to prevent overfitting, which allowed the model to be trained on several train–validation splits. The parameter “k” was equal to 5, which was selected based on a trial-and-error approach. In other words, the data were randomly shuffled and split five times, with 20% of the data used for validation in each fold. The validation error scores were calculated as an average value of all splits.

Training, validation, and testing were performed for 320 combinations. Model performance was evaluated based on the performance indices MSE, RMSE, R^2^, MAE, and NRMSE. The ML models were compared with the linear regression fitting (LR).

Stage 3: Robustness analysis of the five best-performing models

Monte Carlo simulations were used to evaluate how sensitive the models were to the changes in training and testing datasets split. First, five models from Stage 2 with the best performance were chosen. Next, input and output data were randomly split into 80% and 20% parts for training and testing, respectively. The models were then trained again on the randomly split datasets; 800 Monte Carlo simulations were performed per model (4000 simulations in total). Finally, statistical analysis was performed on the results of the MSE and R^2^ for each model to assess the efficiency.

Stage 4. Model interpretation

Feature importance analysis was conducted on one of the models from Stage 3. First, ICE plots and PDPs were calculated for each input variable. In addition, the model was trained with a decreased number of variables to assess the effect of each input on the MSE of the model.

## 6. Results and Discussion

### 6.1. Model Selection

The performance of four ML approaches, i.e., RT, SVM, ET, and ANN, for predicting post-fire compressive strength recovery was compared. Each ML method was trained and optimized by varying the hyperparameters (Table 4) to obtain the lowest possible MSE. Results of the performance indices for the best model obtained for each ML approach are shown in Table 5. The models were compared with linear regression analysis (LR). The values of performance indices for validation represent an average value from the 5-fold cross-validation.

The most accurate Regression Tree (RT) model was obtained for the minimum leaf size 2, with the MSE for the testing dataset equal to 0.0067. In the case of SVM, the cubic kernel function with a kernel size of 3 yielded the lowest MSE, equal to 0.0092. ANN architecture with three hidden layers, with 8, 12, and 12 neurons, respectively, and sigmoid activation function achieved an MSE of 0.0063. The best performance of all the model combinations (MSE = 0.0031) was observed for the Ensemble of Trees with the LSBoost algorithm, minimum leaf size equal to 3, the number of learners equal to 40, and 0.5 learning rate. It is evident that this model also demonstrated the lowest MAE, equal to 0.0424, which is less than 5% of the initial compressive strength, indicating very good accuracy for this prediction.

**Figure 3:**
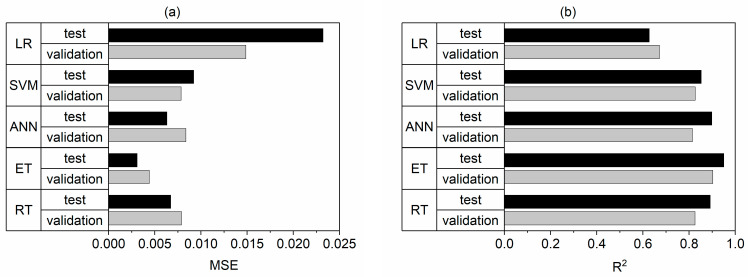
Comparison of the prediction accuracy for the best model within different ML approaches (RT, ET, ANN, and SVM) and linear regression (LR): (**a**) MSE for validation and testing, (**b**) R^2^ for validation and testing.

Error analysis suggested that all analyzed ML approaches had superior accuracy compared to linear regression for training and testing datasets (Figure 3a). 

**Figure 4:**
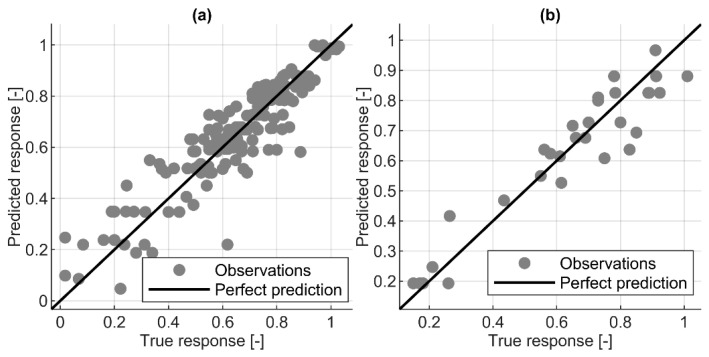

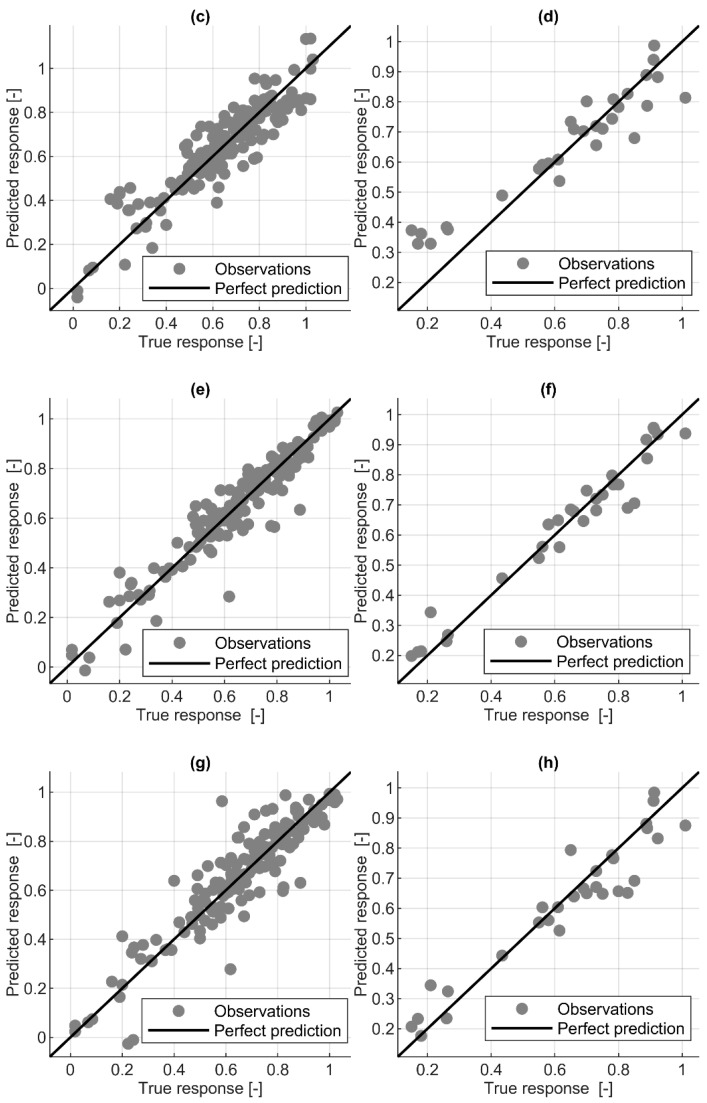

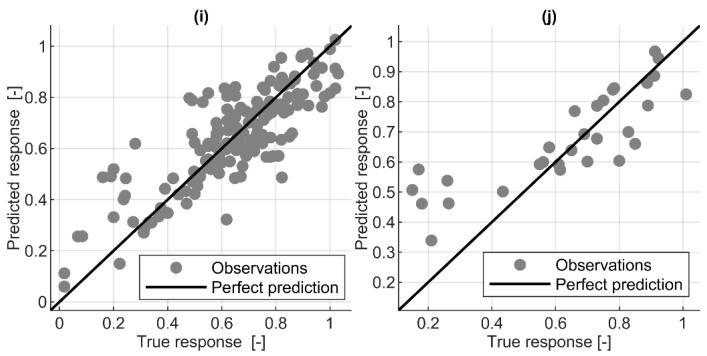
Comparison of the prediction accuracy for the best model within different ML approaches and LR: training and validation: (**a**) RT, (**c**) SVM, (**e**) ET, (**g**) ANN, (**i**) LR; testing: (**b**) RT, (**d**) SVM, (**f**) ET, (**h**) ANN, (**j**) LR.

Overall, the ML MSE was in the range 0.0031–0.0092, which is half that of linear regression, with the MSE of the testing dataset equal to 0.0232. Similarly, the coefficient of determination (R^2^) was less than 0.7 for LR, whereas ML approaches an achieved R^2^ greater than 0.85 for the test dataset (Figure 3). In addition, linear regression obtained an NRMSE equal to 18.8% and 23.4%, which was more than double e.g., the ET model with an NRMSE of approximately 10% (Table 5). All the ML models displayed a moderate linear correlation between predicted and true (measured) strength recovery values, with R^2^ values greater than 0.8 (Figure 4). It is noticeable in some cases that the predicted response values are less than zero. The actual values of the response are positive numbers; nevertheless, the minimum value is very close to zero, equal to 0.018 (Table 2). The proposed model is associated with the error, i.e., with NRMSE between 8–25%, depending on the algorithm (Table 5). Therefore, the predicted values might oscillate around the zero value. This indicates that there is presumably no self-healing for this particular set of predictors.

**Figure 5:**
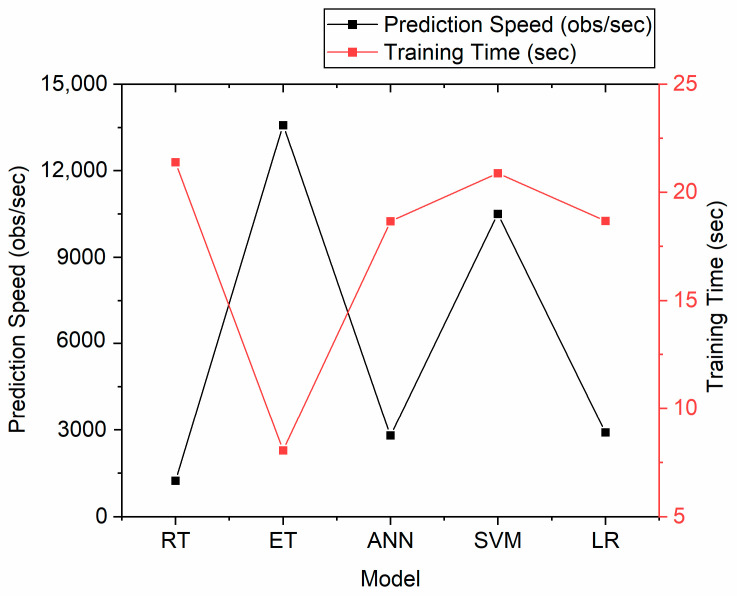
Comparison of the prediction speed and training time for the best model within different ML approaches (RT, ET, ANN, and SVM) and linear regression (LR).

In addition, the models’ prediction speed and training time were compared (Figure 5). The SVM model exhibited a fast prediction speed (approx. 10,000 observations/second) but a relatively long training time (22 s). The slowest in terms of prediction speed was ANN and LR, both with approx. 3000 observations/second. The fastest algorithm (14,000 observations/second) was the ET model, with the shortest training time of approximately 7 s.

Considering the error indices and prediction speed, the ET approach with boosting demonstrated the most accurate and optimal prediction performance. Of all the analyzed model combinations, the five with the lowest MSE error, approximately 0.0045, were the ET models (Table 6). 

**Figure 6:**
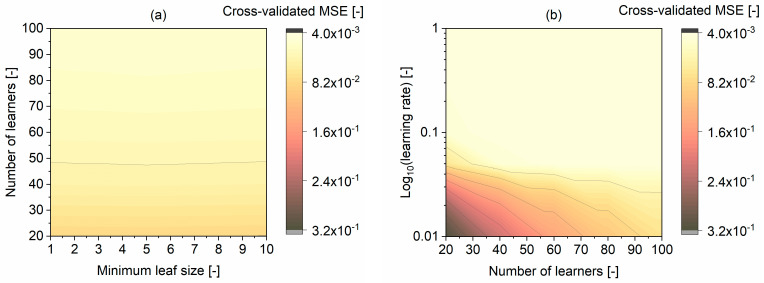
Hyperparameters’ effect on the ET model with the LSBoost algorithm MSE for the training and validation dataset: (**a**) minimum leaf size vs. number of learners, (**b**) number of learners vs. log of the learning rate.

All of those models achieved a comparable MSE of approximately 0.0045 and MAE equal to 0.05, as well as R^2^ greater than 0.9. The models were highly dependent on the learning rate parameter (Figure 6). For the learning rate above 0.5, a smaller number of learners were sufficient to achieve a low MSE. A low learning rate, less than 0.1, was optimal for the number of learners above 80. In the next step, Monte Carlo simulations were performed to assess the selected models’ stability and robustness.

### 6.2. Robustness Analysis

**Figure 7:**
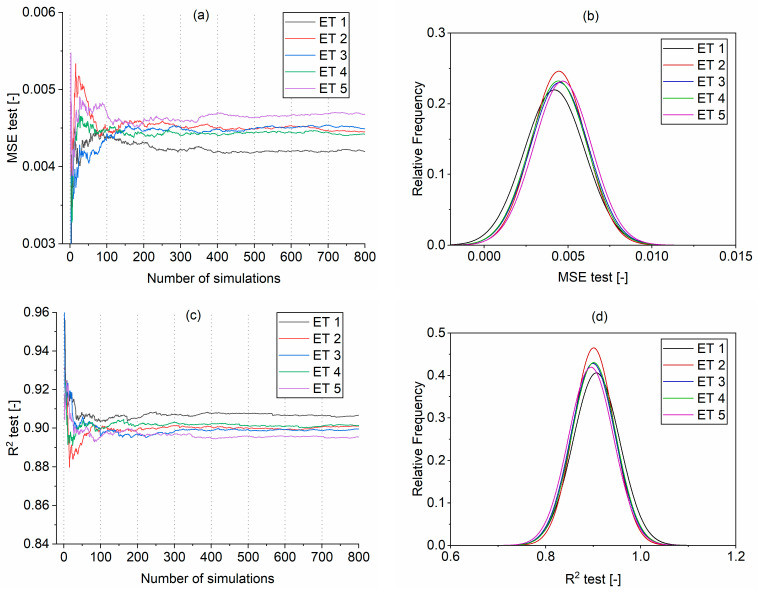
Results of the Monte Carlo simulations for models ET1–ET5: (**a**) changes of MSE for testing dataset, (**b**) normal distribution fitting of MSE, (**c**) changes of R^2^ for testing dataset, (**d**) normal distribution fitting of R^2^.

**Figure 8:**
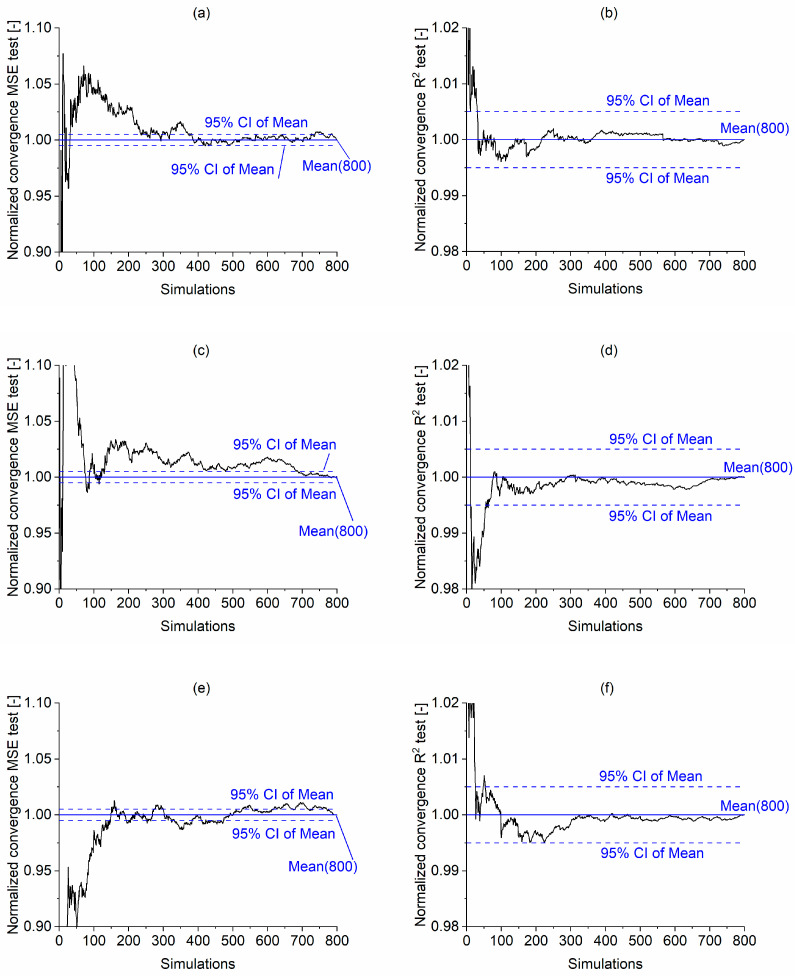

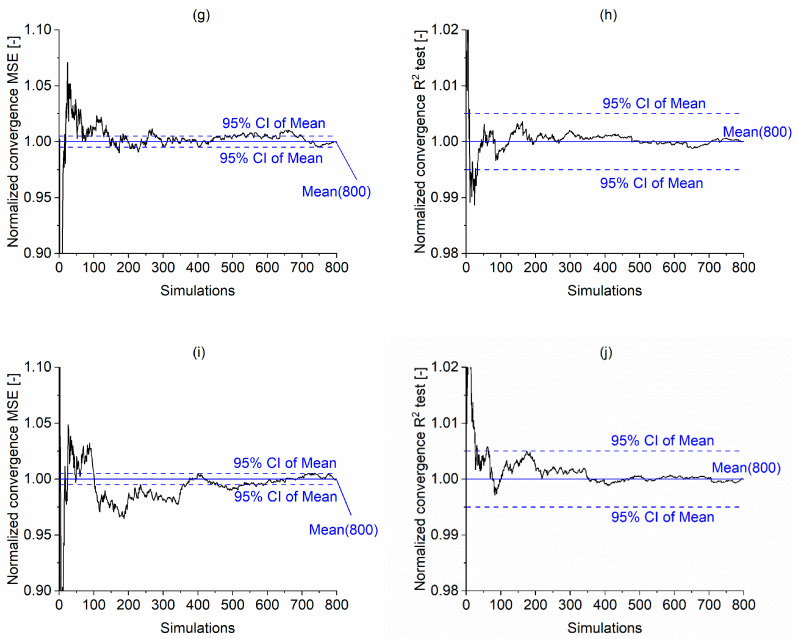
Comparison of the Monte Carlo normalized convergence of testing set MSE and R^2^ for selected models: (**a**,**b**) ET1, (**c**,**d**) ET2, (**e**,**f**) ET3, (**g**,**h**) ET4, (**i**,**j**) ET5.

The robustness analysis was performed using MCS on the models listed in Table 6. The database was split into training and testing parts, comprising 80% and 20%, respectively. No cross-validation was applied. The random sampling effect of the training and testing dataset on the changes of MSE and R^2^ was studied. The initial evaluation suggested that fewer than 800 Monte Carlo realizations seem to warrant a stable solution for the testing dataset (Figure 7a,c). Normal distributions for each model are presented in Figure 8b,d. Model ET1 demonstrated slightly better accuracy than the rest of the models with respect to the mean MSE and R^2^ (Table 7). The standard deviation was comparable for all the models, with values between 0.043–0.049 for R^2^ and 0.0016–0.0018 for MSE (Table 7).

The normalized convergence of testing set MSE and R^2^ calculated according to Equation (5) is shown in Figure 8. The mean value from the 800 realizations and its 95% confidence interval (CI) bounds were marked in blue for each model. It is noticeable that R^2^ values converge only after approximately 100 simulations to the 800-realization average, marked as “Mean (800)”, for all the models (Figure 8b,d,f,h,j). On the other hand, the MSE values display higher variability. For example, for models ET1 and ET4, the convergence (approximately within 95% CI) is achieved at around 350 realizations, while for ET2, it was achieved at 700 realizations, and for ET3 and ET5, 500 realizations.

To reduce the error, more realizations of MCS could be executed; however, the accuracy and robustness of model ET1 are sufficient for the accurate prediction of post-fire self-healing strength recovery.

### 6.3. Model Interpretation

**Figure 9:**
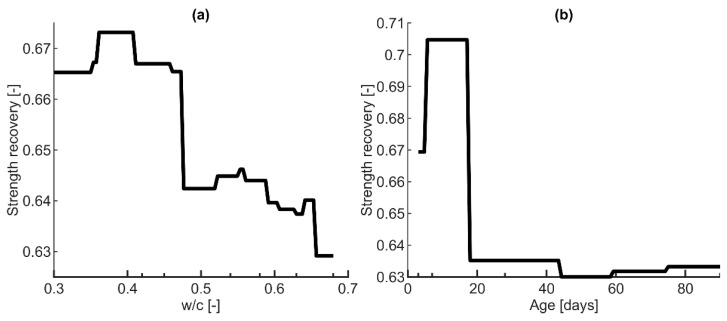

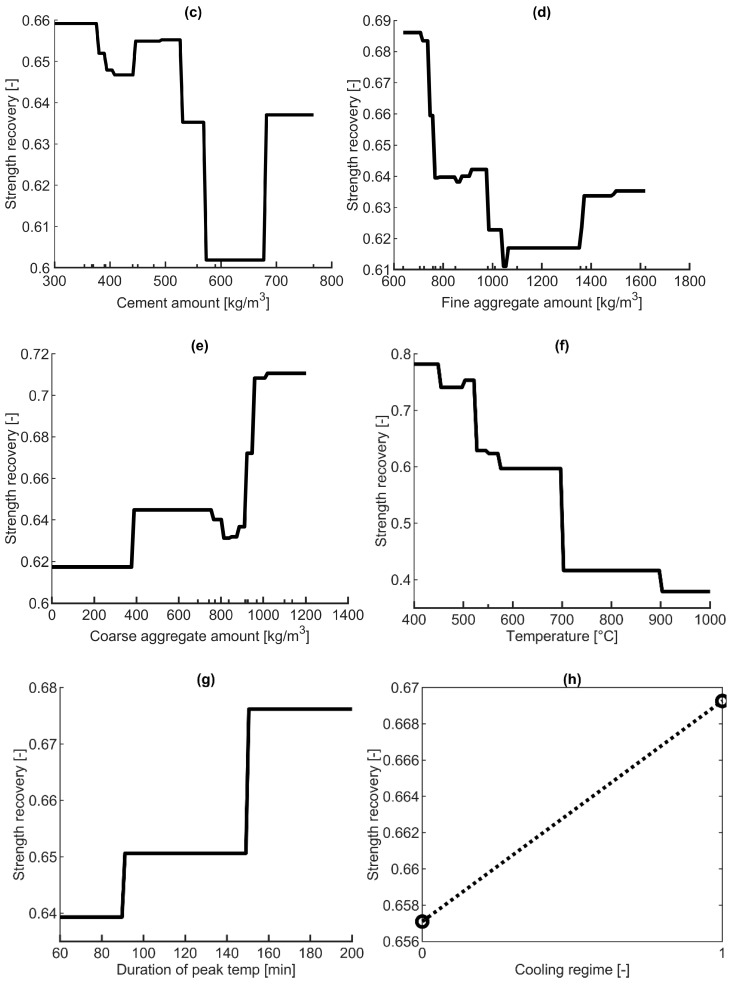

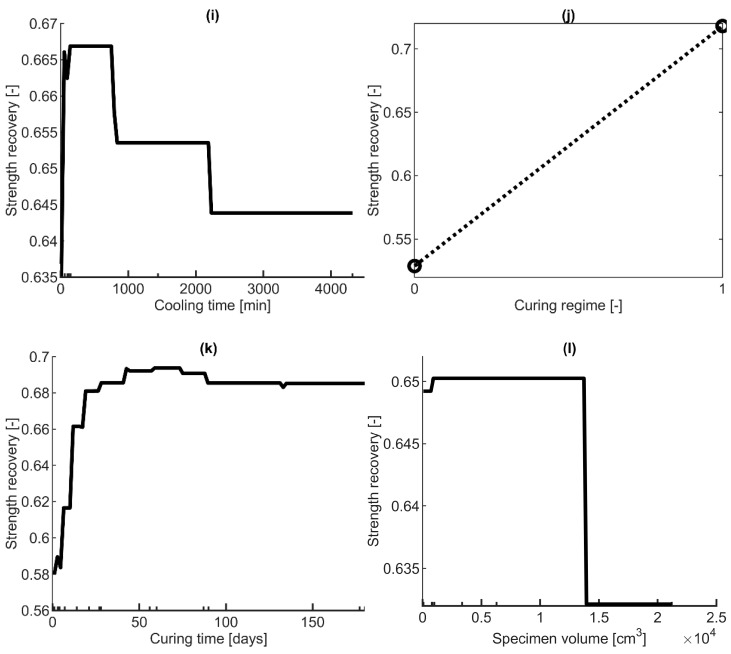
PDPs for each input variable: (**a**) water-to-cement ratio (I1), (**b**) age (I2), (**c**) cement amount (I3), (**d**) fine aggregate (I4), (**e**) coarse aggregate (I5), (**f**) temperature (I6), (**g**) duration of peak temperature (I7), (**h**) cooling regime (I8), (**i**) cooling duration (I9), (**j**) curing regime (I10), (**k**) curing duration (I11), (**l**) specimen volume (I12).

**Figure 10:**
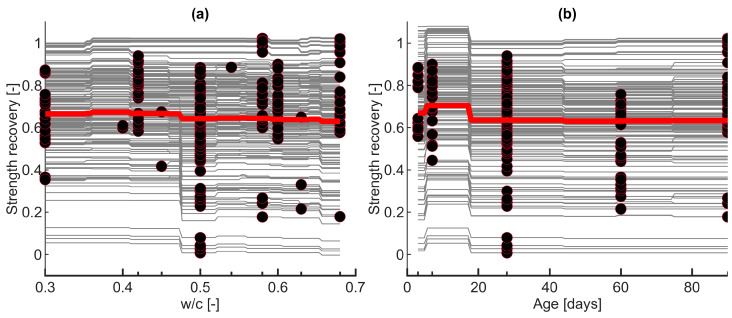

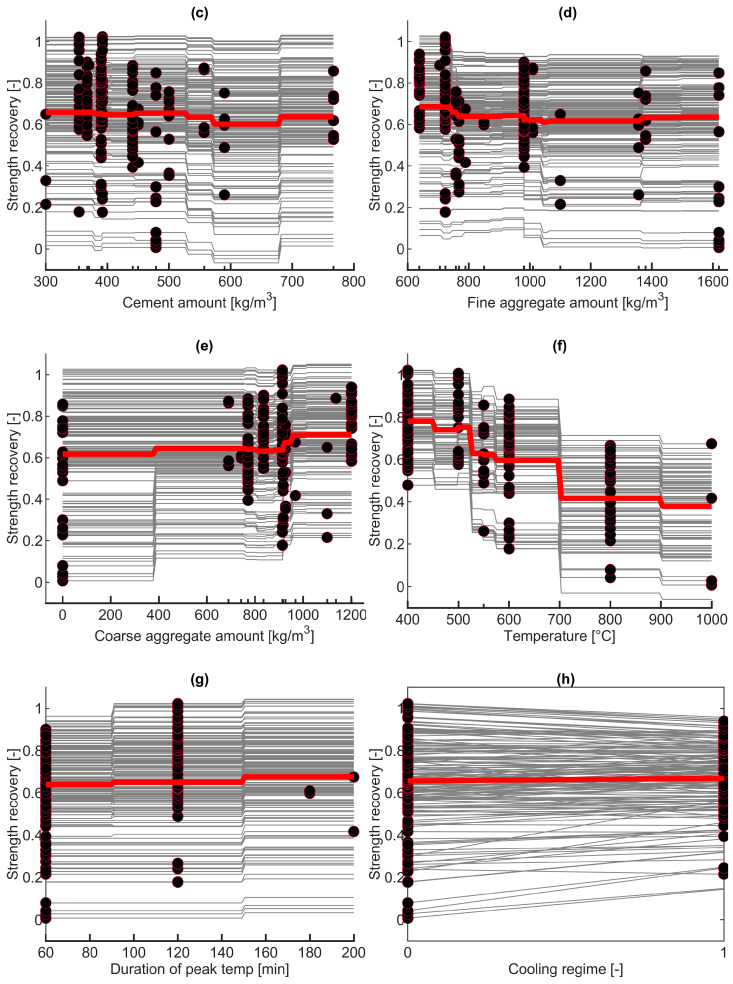

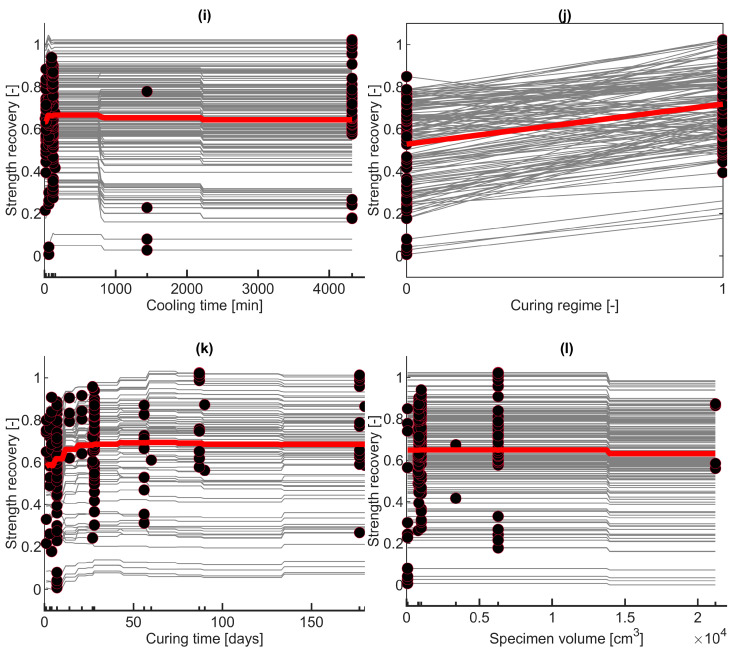
ICE plots for each input variable. The thick red curve depicts the PDP, i.e., the average of all the individual gray curves. Dots represent the measured value of the specific variable. (**a**) Water-to-cement ratio (I1), (**b**) age (I2), (**c**) cement amount (I3), (**d**) fine aggregate (I4), (**e**) coarse aggregate (I5), (**f**) temperature (I6), (**g**) duration of peak temperature (I7), (**h**) cooling regime (I8), (**i**) cooling duration (I9), (**j**) curing regime (I10), (**k**) curing duration (I11), (**l**) specimen volume (I12).

Based on the previous sections, model ET1 (Table 6) was considered for further analysis. This model was used for the variable importance analysis and model interpretation. The PDP (Figure 9) and ICE plots (Figure 10) were calculated for each input variable. It should be noted that the scale in Figure 9 is different for each variable to show the response changes in detail. On the other hand, in Figure 10, the scale is the same for all variables. The red line indicates the PDP as an average of all the curves (Figure 10).

The PDP of the temperature variable demonstrates a significant negative impact of increasing loading temperature on self-healing strength recovery, with the values changing between 0.4 and 0.8 (Figure 9f and Figure 10f). This finding is in good coherence with previous results [7]. It is possibly caused by the chemical and physical changes occurring in concrete at different temperatures. Figure 9f shows two considerable drops in strength recovery at approximately 500 °C and 700 °C. The former can be associated with the decomposition of Portlandite at approximately 400–500 °C, while the latter is associated with the continued disintegration of calcite and calcium silicate hydrate (C–S–H) at 700–900 °C [69]. In addition, with increasing loading temperature, the material has a higher porosity, and the microcracking escalates with the increasing crack width. Wider cracks are more challenging to heal without additional stimulants [70]. Therefore, they cause discontinuities in the cement binder, presumably contributing to an “unrecoverable” compressive strength.

Strength recovery seems to exhibit limited dependency on the concrete’s age (Figure 9b and Figure 10b), with the values ranging between approximately 0.63 and 0.71. There is a slight increase for the early-age concrete, followed by a decrease at approximately 20 days. Afterward, there is no change in strength recovery with respect to the concrete’s age. This observation was confirmed by [43]. Early-age concrete has more unhydrated cement, which in the presence of moisture, further hydrates after temperature exposure, possibly contributing to more efficient post-fire self-healing [43].

It is evident that binder-related variables, i.e., w/c and cement amount, could have a negligible effect on the post-fire strength recovery (Figure 9a,c and Figure 10a,c). This is in agreement with several experimental studies on post-fire healing [47,71], as well as for mechanically cracked concrete healing [72]. The higher the w/c, the lower the strength recovery (Figure 9a); however, the difference in the response is only approximately 5% of the intact specimen’s strength. For the cement amount, there is a noticeable decrease between 500–600 kg/m^3^ of cement, followed by a slight increase for the cement amount above 700 kg/m^3^ (Figure 9c). Nevertheless, the relative difference in strength recovery is less than 5% (Figure 9c and Figure 10c). Comparing the single observation curves on the ICE plot (Figure 10a,c), some inhomogeneities are noticeable, which could mean that both variables could be affected by an interaction with the other predictors [32].

The PDP indicates that specimen volume’s effect is unimportant for the post-fire self-healing strength recovery, causing changes smaller than 1% (Figure 9l). Therefore, the conclusions of the developed model could presumably be also applied to large-scale elements. Some experimental [44,73] and modeling [51] studies also observed a minor dependency of the compressive strength on the specimen size, but no experimental results validated this hypothesis for the post-fire self-healing strength recovery.

The PDP of the cooling regime (Figure 9h) indicates that strength recovery could be causally insensitive to the cooling type, with a change in strength recovery parameter less than 2%. However, the ICE plot (Figure 10h) shows that the cooling regime variable may interact with other predictors, i.e., some curves decrease while others increase. Similar ambiguity was observed from experimental results. For example, cooling caused further compressive strength reduction, while water cooling led to strength recovery [46]; however, water cooling also generated more damage [8]. The cooling time (Figure 9i) has a negative effect on the strength recovery; however, its significance is relatively small, with strength recovery changes of approximately 3–4%. Furthermore, the ICE plot indicates that the effect of the cooling time is roughly additive, i.e., the curves for each observation are parallel to each other [32].

The effect of the curing regime is relatively powerful, with the strength recovery parameter changing between approximately 0.5 and 0.75 (Figure 9j and Figure 10j). Such observation is in agreement with the literature; water curing (here marked as “1”) after high-temperature exposure was found to give the highest strength and durability recovery [10,12,47] in comparison with air curing (here marked as “0”). Nevertheless, in this study, the curing regimes were divided into two groups, i.e., with air and water. The effect could be expected to be even more significant in the case of different types of treatments or a more detailed data split with varied curing categories, e.g., specified relative humidity. 

Similarly, the PDP indicates that strength recovery could be causally sensitive to the curing time for approximately the first 50 days, with the highest self-healing during the first 25 days (Figure 9k). The strength recovery values range between 0.58 and 0.7 for this period. After 50 days, there is a negligible change in the strength recovery, less than 2%. 

**Figure 11:**
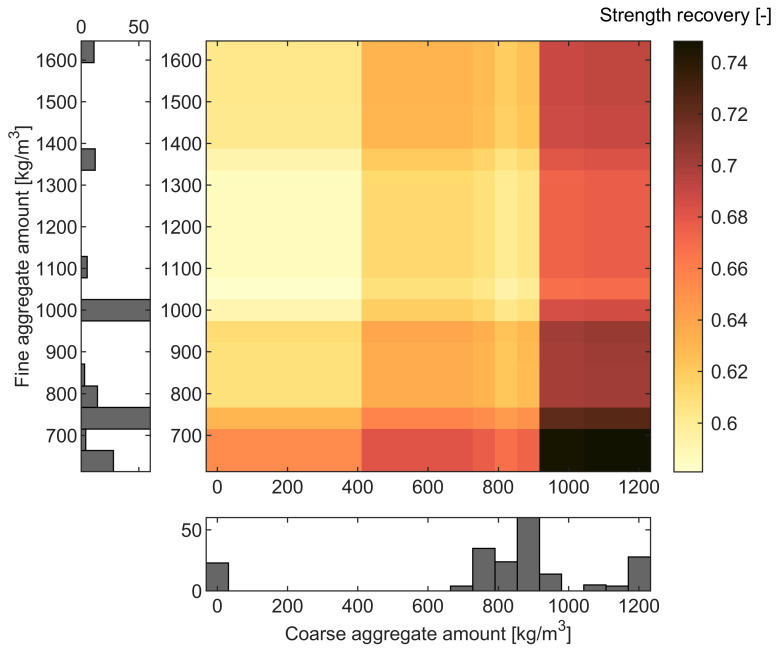
Heatmap PDP for the two variables: coarse and fine aggregate vs. strength recovery. Colors correspond to different values of strength recovery.

The influence of aggregate-based variables, namely, fine (Figure 9d and Figure 10d) and coarse (Figure 9e and Figure 10e) aggregates, is pronounced, with the changes of values in strength recovery between 0.61–0.69 and 0.62–0.72, respectively. There is a notable decrease in strength recovery for fine aggregate until approximately 1000 kg/m^3^, with a slight increase above this value. On the contrary, the amount of coarse aggregate could positively influence the strength recovery. To the authors’ best knowledge, there are no experimental studies on the effect of aggregate types and amounts on post-fire curing. In their review, [6] noted that mortar and concrete generally exhibit better post-fire healing than cement paste. In the case of aggregate-based variables, the recovery results could be attributed to the physical changes under temperature loading, i.e., cracks caused by different thermal expansion coefficients of aggregates [6,74]. These high-temperature cracks form a permeable network which presumably gives more space for the self-healing products [6] and facilitates the transport of moisture and chemical substances into the crack. Analysis of the 2D heatmap PDP for two variables, fine and coarse aggregate (Figure 11), suggests that the most optimal mix composition for the post-fire self-healing strength recovery is a fine aggregate amount of approximately 600 kg/m^3^ and coarse aggregate between 1000–1200 kg/m^3^. However, this relationship can also be affected by these predictors’ high correlation (R = −0.9) (Figure 2).

**Figure 12:**
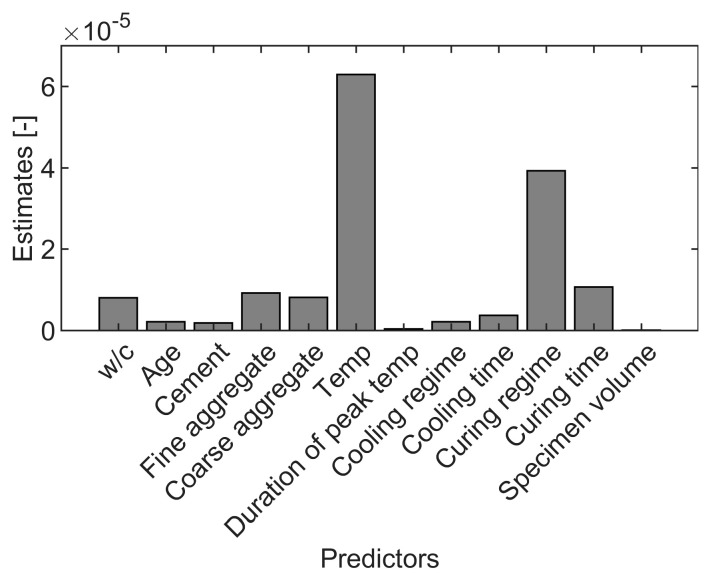
Feature importance results.

In addition to the PDP and ICE plot analysis, the estimates of predictor importance for model ET1 were calculated (Figure 12). The algorithm calculates the sum of changes in the node risk due to splits on every variable. Subsequently, it divides this sum by the total number of branch nodes [75]. The results suggest that the loading temperature, curing regime, and curing time are the most significant input variables. In addition, fine and coarse aggregate also achieved high importance scores (Figure 12). These results agree with the PDP analysis. 

**Figure 13:**
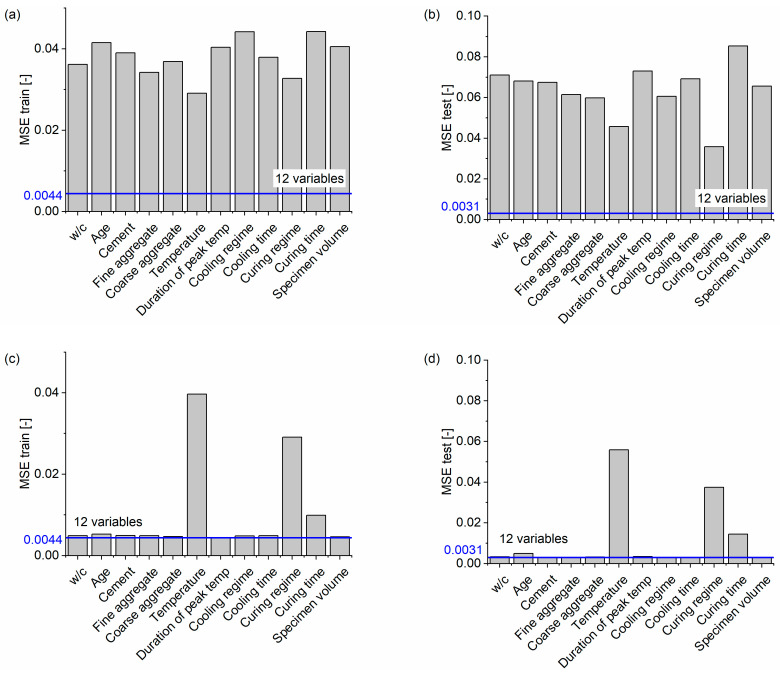
Performance of the model with a different number of variables: (**a**) trained only on one variable, MSE for the training set; (**b**) trained only on one variable, MSE for the testing set; (**c**) trained on 11 variables with one variable removed, MSE for the training set; (**d**) trained on 11 variables with one variable removed, MSE for the testing set.

Another approach to assess the feature importance, which is not dependent on the model type, is measuring the performance drop after retraining with a different number of input variables. Here, two cases were analyzed, one by removing each of the twelve variables and the second by training the model with just one variable. The results of the MSE for those cases for both the training and testing set are presented in Figure 13. Again, it is evident that temperature, curing regime, and curing time show the most significant change in MSE for both analyzed cases, which supports the causal model interpretation performed earlier.

## 7. Conclusions and Future Research

In this paper, for the first time, machine learning modeling was used to predict the compressive strength recovery due to self-healing for the high-temperature damaged concrete. Twelve variables were taken into consideration based on thorough literature studies: w/c, age of concrete, amount of cement, fine aggregate, coarse aggregate, peak loading temperature, duration of peak loading temperature, cooling regime, duration of cooling, curing regime, duration of curing, and specimen volume. The post-fire compressive strength recovery prediction was built with four ML approaches, i.e., SVM, ANN, RT, and ET. An exhaustive analysis of the model was performed using PDP and ICE plots. The following major conclusions can be drawn from this study:

- All four ML approaches demonstrated higher accuracy than linear regression in terms of MSE, MAE, RMSE, and R^2^. Optimized ET with boosting achieved the best performance concerning prediction precision (NRMSE of approximately 10% and R^2^ greater than 0.9) and speed. The model showed a high dependency on the learning rate. The robustness analysis with the use of MCS confirmed the stability of the model’s prediction capacity.
- Prediction analysis revealed that temperature, curing regime and curing time, and aggregates’ amounts are the critical input variables. Mix composition parameters, such as cement amount and w/c ratio, presumably play a secondary role in the healing mechanism. Nevertheless, additional experiments should be performed to confirm this relationship.
- The model indicated that the optimal amount of fine and coarse aggregate to achieve strength recovery greater than 74% is presumably equal to 600 kg/m^3^ and 1000–1200 kg/m^3^, respectively. Water exposure was found to be the most efficient. The curing was significant only during the first 50 days of healing.

The study showed that ensemble ML algorithms could successfully predict the post-fire self-healing of concrete. Furthermore, the causal interpretation performed using ICE plots and PDPs suggested that future experiments on self-healing of thermally cracked concrete could focus on improving the curing treatment since it had a significant effect on strength recovery. The model can be potentially applied to solve an inverse problem, i.e., optimizing concrete mix composition to obtain a high compressive strength recovery. Nevertheless, the following limitations and potential advancements can be proposed for this study:

- This paper assumes only compressive strength recovery as the post-fire healing response. Analyzing other outputs, such as crack closure and durability recovery, could give further insights into the recovery mechanism. However, there are sparse data regarding these parameters in the literature; therefore, more experimental work should be conducted.
- Performed analysis was limited to only two types of curing and cooling regimes, i.e., air and water. Other types of curing and cooling regimes could be “encoded” with categorical variables, e.g., wet–dry cycles, different levels of RH, etc.; the effect of cement replacement with supplementary cementitious materials should be studied to verify the binder blending importance on the post-fire self-healing. To date, there are limited results on this topic; therefore, the database should be expanded.
- Additional sensitivity analysis could be performed using Monte Carlo simulations with permutation feature importance [63]. Finally, the effect of multicorrelations between input variables should be studied as it might obscure actual relationships between inputs and output. A reduction of the number of predictors should be considered.

## Figures and Tables

**Table 1:** Examples of ML modeling of autogenous self-healing.

References	ML Algorithm	Database Size	Inputs	Outputs	Accuracy	Pros	Cons
[36]	Ensemble methods: AdaBoost regressor, Decision Tree, and bagging regressor	617	Fly ash, limestone powder, silica fume, and initial crack width	Crack width after healing	R^2^ = 0.974 for bagging	Good prediction accuracy Parameter importance analysis performed	Applied only to engineered cementitious composites A small number of inputs Output does not consider durability/mechanical performance Self-healing exposure is not considered
[37]	Artificial Neural Network with hybrid genetic algorithm	1462	Cement content, w/c, type and dosage of supplementary cementitious materials, bio-healing materials, expansive and crystalline additives	Crack width after healing	R^2^ > 0.99	Excellent accuracy Many inputs Large database	Output does not consider durability/mechanical performance Self-healing exposure is not considered
[33]	Artificial Neural Network with	2786	Cement content, w/c, supplementary cementitious materials content, fiber content, initial crack width, age of treatment	Self-healing index, including a combination of crack size, permeability, ultrasonic pulse velocity, and mechanical strength changes before and after healing	R^2^ = 0.78 (validation and testing dataset)	Model analysis Comparison with meta-analysis results Extensive database Output includes several parameters	Relatively low accuracy Self-healing exposure is not considered
[35]	Artificial Neural Network, k-nearest neighbors, gradient boosting regression, decision tree regression, Support Vector Regression, and a Random Forest	1417	Type and dosage of healing materials, fiber diameter, length and tensile strength, initial crack width, the time for healing, cracking age, the healing condition, cement type and content, superplasticizer, fine aggregates, fly ash and slag content, w/b	Crack width after healing and resonance frequency	R^2^ = 0.958	Good prediction accuracy Many inputs Large database Parameter importance analysis performed Includes exposure conditions	Output does not consider durability/mechanical performance
[34]	Artificial Neural Network, Support Vector Machines, Classification and Regression Tree, with ensemble: bagging, AdaBoost, and stacking	617	Fly ash, limestone powder, silica fume, and initial crack width	Crack width after healing	R^2^ = 0.90 for bagging	Training on consistent experimental data Comparison of ensemble and individual models	Applied only to engineered cementitious composites A small number of inputs Output does not consider durability/mechanical performance Self-healing exposure is not considered

**Table 2:** Sources used for the database construction.

No.	References	Samples in Dataset
1	[10]	2
2	[43]	56
3	[44]	3
4	[11]	4
5	[12]	8
6	[45]	4
7	[46]	11
8	[47]	48
9	[8]	5
10	[48]	24
11	[49]	4
12	[50]	28
	Total	197

**Table 3:** Statistical descriptors of the input and output parameters.

Input/ Output	Name	Unit	Min	Max	Median	Mean	Std	Sk
I1	w/c	-	0.3	0.68	0.5	0.51	0.11	0.37
I2	Age	days	3	90	28	43.65	30.70	0.45
I3	Cement	kg/m^3^	300	767	392	422.80	84.87	2.24
I4	Fine aggregate	kg/m^3^	638.04	1620	768	896.20	257.66	1.42
I5	Coarse aggregate	kg/m^3^	0	1201.59	914	818.98	329.04	−1.56
I6	Peak loading temperature	°C	400	1000	600	569.04	148.65	0.70
I7	Duration of peak loading temperature	min	60	200	120	92.49	33.51	0.44
I8	Cooling regime	-	0	1	-	-	-	-
I9	Duration of cooling	min	10	4320	120	1146.50	1816.16	1.17
I10	Curing regime	-	0	1	-	-	-	-
I11	Duration of curing	days	1	180	27	34.33	45.72	2.22
I12	Specimen volume	cm^3^	64	21,205.8	1000.0	2823.5	3616.9	2.8
O	Recovered compressive strength	-	0.018	1.03	0.66	0.65	0.22	0.74

**Table 4:** Studied models’ hyperparameters.

Algorithm	Support Vector Machine	Regression Tree	Ensemble of Regression Trees	Artificial Neural Network
Parameters of the algorithm	Kernel function (Gaussian, linear, cubic, quadratic) Kernel scale (1–15) Box constraint—constant Epsilon—constant	Minimum leaf size (1–15)	Boosted/Bagged Minimum leaf size (1–10) Number of learners (20–100) Learning rate (0.01–1)	Number of layers (1–3) Number of neurons in the layer (2–12) Activation function (ReLu, tansig, sigmoid)

**Table 5:** Performance of the most accurate models for each ML approach.

ML Approach	Best Model Parameters	Dataset	MSE (-)	MAE (-)	R^2^ (-)	RMSE (-)	NRMSE (%)
RT	Min. Leaf size 2	Validation	0.0079 *	0.0651 *	0.826 *	0.0889 *	13.7 *
		Testing	0.0067	0.0667	0.892	0.0821	12.6
SVM	Cubic kernel, Kernel size 3	Validation	0.0078 *	0.0672 *	0.827 *	0.0886 *	13.6 *
		Testing	0.0092	0.0731	0.852	0.0960	14.8
ET	LSBoost algorithm, Min. Leaf size 3, Number of learners 40, Learning rate 0.5	Validation	0.0044 *	0.0448 *	0.903 *	0.0664 *	10.2 *
		Testing	0.0031	0.0424	0.950	0.0557	8.6
ANN	Layers 8:12:12, Activation function: sigmoid	Validation	0.0084 *	0.0617 *	0.815 *	0.0915 *	14.1 *
		Testing	0.0063	0.0598	0.899	0.0795	12.2
LR	-	Validation	0.0149 *	0.0914 *	0.672 *	0.1219 *	18.8 *
		Testing	0.0232	0.1119	0.628	0.1524	23.4

* The value is an average from 5-fold cross-validation.

**Table 6:** Prediction performance of five best-performing models.

Model	Model Parameters	Dataset	MSE (-)	MAE (-)	R^2^ (-)	RMSE (-)	NRMSE (%)
ET1	LSBoost algorithm, Min. Leaf size 3, Number of learners 40, Learning rate 0.5	Validation	0.0044 *	0.0448 *	0.903 *	0.0664 *	10.2 *
		Testing	0.0031	0.0424	0.950	0.0557	8.6
ET2	LSBoost algorithm, Min. Leaf size 5, Number of learners 100, Learning rate 0.1	Validation	0.0044 *	0.0483 *	0.903 *	0.0662 *	10.2 *
		Testing	0.0048	0.0519	0.923	0.0692	10.6
ET3	LSBoost algorithm, Min. Leaf size 3, Number of learners 20, Learning rate 0.5	Validation	0.0044 *	0.0463 *	0.902 *	0.0666 *	10.2 *
		Testing	0.0043	0.0476	0.931	0.0657	10.1
ET4	LSBoost algorithm, Min. Leaf size 2, Number of learners 100, Learning rate 0.1	Validation	0.0045 *	0.0485 *	0.901 *	0.0669 *	10.3 *
		Testing	0.0045	0.0491	0.928	0.0669	10.3
ET5	LSBoost algorithm, Min. Leaf size 5, Number of learners 80, Learning rate 0.1	Validation	0.0045 *	0.0492 *	0.901 *	0.0671 *	10.3 *
		Testing	0.0053	0.0543	0.915	0.0728	11.2

* The value is an average from 5-fold cross-validation.

**Table 7:** Summary of error criteria of the MCS for the testing dataset.

Model	R^2^ (-)				MSE (-)			
	Min	Max	Mean	Std	Min	Max	Mean	Std
ET1	0.437	0.979	0.907	0.049	0.0012	0.013	0.0042	0.0018
ET2	0.690	0.974	0.901	0.043	0.0010	0.012	0.0045	0.0016
ET3	0.537	0.977	0.900	0.047	0.0012	0.012	0.0045	0.0017
ET4	0.585	0.970	0.901	0.046	0.0016	0.015	0.0044	0.0017
ET5	0.512	0.972	0.895	0.048	0.0016	0.012	0.0047	0.0017

## Data Availability

The data presented in this study are available on request from the corresponding author.
